# Discovering Suspicious APT Behaviors by Analyzing DNS Activities

**DOI:** 10.3390/s20030731

**Published:** 2020-01-28

**Authors:** Guanghua Yan, Qiang Li, Dong Guo, Xiangyu Meng

**Affiliations:** 1 College of Computer Science and Technology, Jilin University, Changchun 130012, China; yangh17@mails.jlu.edu.cn (G.Y.); li_qiang@jlu.edu.cn (Q.L.); 2 Key Laboratory of Symbol Computation and Knowledge Engineering, Jilin University, Ministry of Education, Changchun 130012, China; guodong@jlu.edu.cn

**Keywords:** APT attack, DNS, deep learning, behavior detection, sensor network

## Abstract

As sensors become more prevalent in our lives, security issues have become a major concern. In the Advanced Persistent Threat (APT) attack, the sensor has also become an important role as a transmission medium. As a relatively weak link in the network transmission process, sensor networks often become the target of attackers. Due to the characteristics of low traffic, long attack time, diverse attack methods, and real-time evolution, existing detection methods have not been able to detect them comprehensively. Current research suggests that a suspicious domain name can be obtained by analyzing the domain name resolution (DNS) request to the target network in an APT attack. In past work based on DNS log analyses, most of the work would simply calculate the characteristics of the request message or the characteristics of the response message or the feature set of the request message plus the response message, and the relationship between the response message and the request message was not considered. This may leave out the detection of some APT attacks in which the DNS resolution process is incomplete. This paper proposes a new feature that represents the relationship between a DNS request and the response message, based on a deep learning method used to analyze the DNS request records. The algorithm performs threat assessment on the DNS behavior to be detected based on the calculated suspicious value. This paper uses the data of 4, 907, 147, 146 DNS request records (376, 605, 606 records after DNS Data Pre-processing) collected in a large campus network and uses simulation attack data to verify the validity and correctness of the system. The results of the experiments show that our method achieves an average accuracy of 97.6% in detecting suspicious DNS behavior, with the orange false positive (FP) at 2.3% and the recall at 96.8%. The proposed system can effectively detect the hidden and suspicious DNS behavior in APT.

## 1. Introduction

With the development and popularity of the Internet of Things today, sensor networks are widely used in people’s daily lives. The term Wireless Sensor Network (WSN) refers to a group of spatially dispersed and dedicated sensors for monitoring and recording the physical conditions of an environment and organizing the collected data at a central location. WSNs measure environmental conditions like temperature, sound, pollution levels, humidity, wind speed and direction, pressure, etc., [1]. In the process of data transmission, the vulnerability of the software layer has become one of the major concerns of sensor network security. In the report of Reference [2], the attackers use the Internet of Things to attack the target. They take advantage of weak passwords in cameras and routers to gain access to hosts and install malware in them [3]. Among many attacks, Advanced Persistent Threat (APT) attacks as a kind of high-concealed, high-profile attacks have naturally become a major threat to sensor networks.

APT is a new type of network attack, which can freely use multiple attack techniques. APT persistently collects data from a specific target by exploiting vulnerabilities using diverse attack techniques. Although many new methods have been deployed to detect APT attacks, the type and number of new APT attacks are still increasing [4].

APT attacks are targeting and hidden. Attackers usually investigate the targets in advance and try to get their private information. Later, they will try to infect the targets with various customized attacking methods, such as phishing, waterhole attack, and zero-day attack. Once the targets are infected, attackers will further compromise the targets with some novel malware [4]. Since the novel malware is not discovered in the wild yet, its signature and behavior will easily bypass anti-virus tools. The popular techniques used in the malware, such as packing and obfuscation, make the detection even harder [5]. Once the target is fully compromised, attackers can stealthily collect the sensitive data by mixing their traffic with normal network traffic [4], using the tunneling technique to avoid the detection for anomaly network [6], encrypting the packets, making up valid file signature, and communicating in a low frequency.

The network communications between the infected user and the attacker occur multiple times in the whole attacking process, including initial infection, further infection, and data collection. Traditional detection methods for anomalous network communication can be easily avoided by existing APT attacks [7,8]. However, these communications will inevitably generate the related DNS request and response activities. Several works have demonstrated the possibility of APT attack detection by analyzing DNS activities [9,10].

The existing works usually extract some features that can show the relationship between IP and domain. They only analyze the static features and static associations in the DNS request and response message. Such features can be easily avoided by using DNS tunneling [9], which has been proven able to avoid the firewall and Intrusion Detection System [6,11]. Motivated by this, we analyzed the relationship between APT attacks and the DNS.

For this paper, we designed a new system to detect APT attacks based on DNS logs. By doing a deep analysis of our collected logs, we found seven DNS features that are strongly connected with suspicious APT attacks. Motivated by the recent advance in machine learning, we designed a neural network to train an effective model to find the relationship between DNS activities and APT attacks. The experiment results based on a large dataset demonstrated the effectiveness of our model in detecting APT attacks, with a recall of 96.8% and an accuracy of 97.6%.

The main contributions of this paper are as follows:

- We found seven features, which are classified into three categories, and eight features that are related with DNS logs in APT activities. We proposed three new features related to DNS behaviors, which are the relationships between DNS request message and the DNS response message. We also presented a new way to express the temporal characteristics of a DNS log.
- We proposed a new system to detect DNS malicious behavior and to analyze DNS logs to generate the feature set. We used deep learning to do research.
- We verified the validity of our detection system, and the results of the experiments show that our method achieves an average accuracy of 97.6% in detecting suspicious DNS behavior, with the false positive (FP) at 2.3%, and recall at 96.8%, which shows that our system can detect the malicious DNS behaviors in APT effectively.

## 2. Related Work

### 2.1. Attacking Models of APT

Attacking models of APT are of more types and of more specific targets than traditional ones, and the elements they attack are of a wide variety.

Martin Ussath et al. found in their research [12] that the main characteristics of APT attacks are (1) attack specific targets, (2) use sophisticated tactics, techniques and procedures, (3) constantly evolve their attacking steps, (4) largely infiltrate into a network, (5) perform repeating attacking attempts, and (6) maintain long-term access to target environment. They divide an APT attack into three stages: Initial Compromise, Lateral Movement, and C&C.

Meicong Li et al. divide an APT attack into four stages [13]: Preparation, Access, Resident, and Harvest. In [14], on the prediction and analyses of APT attacks, Saurabh Singh et al. express their belief that attacks can form an attack cycle composed of Reconnaissance, Breach, Infiltration, Exfiltration, and Stealth persistence.

Artur Rot et al. gave a detailed description of an attack cycle [15] which consists of define target, find and organize accomplices, build or acquire tools, research target infrastructure/employees, test for detection, deployment, initial intrusion, outbound connection initiated, expand access and obtain credentials, strengthen foothold, exfiltrate data, cover tracks, and remain undetected.

**Figure 1:**
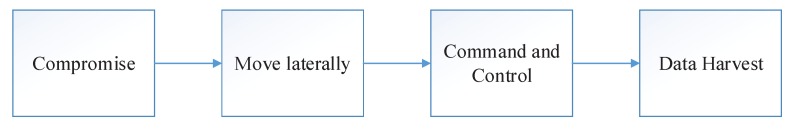
Advanced Persistent Threat (APT) attack model.

Security personnel thoroughly studied the process of APT attack. Reference [11,12], as well as the report of FIREEYE in Reference [4], explain the process and means of APT attacks in detail. In the research on the APT attack stages listed above, the author has divided its entire life cycle in detail at a macro level. We are concerned about the response of APT attacks to DNS behavior. Therefore, the research on the attack method of the attacker during the entire APT attack cycle is unnecessary. The purpose of each phase we distinguish is independent. And at each stage, the DNS behavior we study will respond to the innocent nature of its behavior. We make the following summary(see in Figure 1) to divide an APT attack into four stages [16].

(1) Compromise: Compromise is the first contact between an attacker and its victim. As the start of the entire attack process, it is targeting and deceptive. Here are some typical attack methods: waterhole attack; mail attachment; phishing mail; DDOS attack; and using intermediate system virus infection to assist storage poisoning.

(2) Move Laterally: In this stage, Trojan or malware will generally use the following attack methods to get the resources needed for the attack, like Interface or permission; remote control; exploiting vulnerabilities; SQL injection; buffer overflow; technology of e-picture hiding malicious code.

(3) Command and Control: The target host communicates with the attacker’s C&C server. In this process, http or https communication will be generated for communication, based on which orange File Transfer Protocol (FTP) communication may be used to filter out target sensitive data. Usually the C&C server is a well-hidden server of a dynamic domain name’s IP, which means it is very difficult to track.

(4) Data Harvest: After stealing the data at the stage of Command and Control, attackers will use some means to bring the data back to the network domains which are under their control. Attackers can use the following ways to make the mission accomplished: bring data back through the net bridge of dark network, bring data back via the network disk, or use Trojan to bring data back.

### 2.2. Existing APT Detection Methods

In a monitoring system called Segugio [17], the authors proposed a monitoring algorithm based on a bipartite graph of query relationship, which can track unknown maliciously controlled domain names in the internal network. Segugio generates an IP-DOMAIN undirected bipartite graph from a large amount of DNS data, then marks the nodes in the graph to extract the characteristics of the graph, and uses the machine learning method to detect unknown maliciously controlled domain names. But there is no analysis of the characteristics of the long duration (we define long duration as over 28 days) of APT attacks.

Oprea Alina et al. proposed a new framework based on belief propagation of graph theory to solve early detection problems of business infections [13]. Belief propagation is built on infected hosts provided by professionals, and then a C&C communication detector is established to detect communication between the host and the C&C server. Finally, suspicious domain names are output through a belief propagation algorithm. The framework only detects destination addresses that have never been accessed or rarely accessed, greatly decreasing the data, which is the main shortcoming of the framework. So, attackers can take specific techniques and strategies to avoid its detection.

Zhao et al. proposed a systematic framework called IDNS [7], which uses DNS analysis technology to detect suspicious C&C domain names and then establishes a reputation evaluation engine for calculating the reputation score of the IP address to be detected by using signature-based and anomaly-based detection technique to analyze the traffic related with suspicious IPs. Analysis of DNS logs is an important part of the framework. In the framework, the features of DNS are defined as inherent features of domain names or time features of accessing which do not contain the relationship between the request message and the response message we mentioned before.

Mirco Marchetti et al. [14] detected hosts with suspicious behavior by analyzing a large number of host features. Yong Shi et al. [10] used principal component analysis, k-means clustering, and outlier detection based on median absolute bias. Researchers have used the injected attack traffic in a real enterprise’s network datasets to assess the accuracy of identifying infected hosts against various attack communication models. Reference [15] proposed a Targeted Complex Attack Network (TCAN) model of APT attacks based on dynamic attack graphs and network evolution. The attack process is simulated based on the dynamic evolution rules of complex network theory and actual attack scene features.

In the research of Johnson et al. [18], the authors proposed a novel graph analytic metric that can be used to measure the potential vulnerability of a cyber network to specific types of attacks that use lateral movement and privilege escalation, such as the well-known Pass the Hash, (PTH). The metric can be calculated dynamically from the authorization and auditing layers during the network security authorization phase and will potentially enable predictive de terrence against attacks, such as PTH.

Li et al. studied APT attacks in Hong Kong and the malware function [19]. The researchers investigated the APT attack process by analyzing malware Operation Shady RAT.

Hu et al. simulated the APT by using the game model to identify the best response strategies for each player and proved the existence of Nash Equilibrium for both games [20]. Extensive numerical study further verifies our analytic results and examines the impact of different system configurations on the achievable security level.

McLaren et al. studied for a deeper analysis of features which can reliably identify the establishment of botnet and advanced persistent threat command and control channels [21]. This is achieved by a review of major research, analysis of feature classes, and identification of publicly available sources of benign and malware network traffic.

Begleiter et al. presented a fast and scalable method for detecting threats in large-scale DNS logs [22]. With their method, a language model algorithm learns normal domain-names from a large dataset to rate the extent of domain-name abnormality within a big data stream of DNS queries in the organization.

In some cases, attackers can contact the victim through DNS-Tunneling, through which the response message is not generated in DNS logs, and only the request message is generated. In the DNS resolving process, the external server can also be analyzed as a research object. Attackers often attack the server through an external DNS server, which enhances the concealment of the attack. Prior works based on DNS behavior analysis did not establish the relationship between response message and request message but simply calculated the time features of the request message or the time features of the response message or the inherent features of domain names in DNS behavior. Some APT attacks with incomplete DNS process may be missed through such simple analyses.

## 3. Overview of the Approach

### 3.1. Extrating Features for Detection

The detection framework in our paper is to detect the DNS behavior of malicious behavior that may be used for APT attacks. For this purpose, we analyzed data from 4,907,147,146 DNS request records (376,605,606 records after DNS data pre-processing) collected from the large campus network from 1 April 2018, to 17 May 2018. Through the reading analysis of a large number of APT reports, we summarized the features of DNS behavior in APT attacks and analyzed the features existing in DNS behavior. Through the analysis of the huge data mentioned above, we implemented a detection framework that can define a suspicious value of DNS behavior exploited by APT attacks.

### 3.2. Architecture of the System

**Figure 2:**
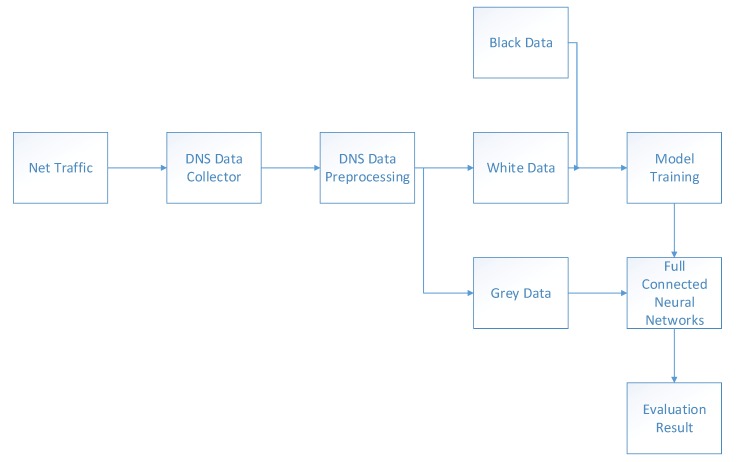
Architecture of the system. DNS = domain name server.

Figure 2 shows the architecture of the system, which consists of four main units: DNS data collector, DNS data pre-processing, DNS behavior feature extraction, and DNS behavior analysis and evaluation (deep learning algorithm).

• DNS Data Collector

It is located in the network data center and records the DNS request behavior and response behavior in the form of logs.

• DNS Data Pre-Processing

Based on the features used in the analysis and evaluation in our system, this unit is divided into two parts: data’s preliminary feature generator and data’s pre-processing filter. Data’s preliminary feature generator: In order to detect whether subsequent features in the system can be extracted or not, we generate a few features for pre-processing and feature extraction. Data’s pre-processing filter: There are two prerequisites for filtering. The first one is whether the components of the domain name set resolved by the DNS behavior record contain the top-level domain names in the whitelist. The second one is that a host resolves a domain name no more than θ times. The most important traffic characteristics of an APT attack is low traffic and long duration. If a domain name is resolved too many times in a certain period of time, we will not regard it as an APT attack.

• DNS Behavior Feature Extraction

This unit extracts the features of the pre-processed DNS behavior record. These features are used to analyze and evaluate the suspiciousness by deep learning algorithm.

• DNS Behavior Analysis And Evaluation

It aims to get a reputation score for a DNS behavior by analyzing feature vectors to decide whether it is used by APT attackers.

## 4. Malicious DNS Features

In this paper, we define eight features for detecting DNS malicious behavior in APT attacks based on a large number of reports and a large amount of data analysis (see Table 1). Three of these features are not mentioned in previous studies. At the same time, we also give a new understanding of the old features. In this section, we will detail the eight features used to detect APT’s malicious DNS behavior.

### 4.1. Inherent Features of Message

(1) Length of a Domain Name Resolved: In APT attacks, an attacker typically sets a malicious domain name at a random length. However, we believe that for some malicious domain names disguised as innocent domain names, their camouflage has certain features. In some malicious domain names, functional words, such as mail, news, update, or some seemingly innocent domain names, will be added to a domain name to form a malicious domain name with a fishing tendency. Due to such a domain name’s construction process, we may be able to determine the maliciousness of a domain name by the length of it.

(2) Number Features of a Domain Name Resolved: Several types of malicious domain names with numbers have been found: A malicious domain name with a number after or before a regular domain name. Attackers add a number when a malicious domain name is pretending to be a regular domain name, such as ‘o’ may be replaced with ‘0’, or ‘I’ may be replaced with ‘1’. Sometimes attackers do not want the domain names to direct to the C&C server, so they usually change the domain names to direct to some specific IPs. Specific IP addresses are usually as follows: 127.0.0.1 (loop back address); 192.168.x.x, 172.16.x.x, 10.x.x.x (private address); x.x.x.255 (broadcast address) [23,24].

Pre-defined IP: Some advanced malware in APT attacks have improved this method. When attackers were developing and coding advanced malware, a predefined IP was hard-coded into the malware’s binary file. When DNS needs resolving, malware will switch to a silent mode, in which the system would not initiate a connection but resolve the domain name to another IP address. Predefined IP addresses are usually some valid IP addresses with obvious features, such as 5.5.5.5, 2.3.3.2. After analyzing the characteristics of the domain name in the above attack situation, it is not difficult to conclude that the number in the domain name can be analyzed as the domain name characteristics in the malicious attack [9].

(3) Features of Keywords in Domain Names: (a) In order to convince the victim that the visited domain name is secure, attackers often disguise the malicious domain names as a famous website. For example, ‘taobao’, ‘alipay’, and other famous websites’ domain names are added to malicious domain names. (b) The other kind of key words used to disguise malicious domain names are functional keywords, such as ‘mail’, ‘news’, ‘update’, etc.

### 4.2. Time Features

Due to the different stages of an APT attack and the concealment of traffic, the time of network traffic has the features of discontinuity and low frequency.

(4) The Frequency of Continuously Resolving the Same Domain Name In a Period of Monitoring Time: Due to the different stages of an APT attack, suspicious traffic will not be generated during the stages. This characteristic is evident in the stage of lateral movement. Once the malware or Trojan gets the permission and the resources that attackers want to get, they will generate communication with the C&C server. This communication process may consist of multiple requests.

(5) Whether the Resolved Domain Name Only Appears Once: We need to discuss this feature from the following two aspects. (a) For the malware or Trojan installation in the initial compromise stage of an APT attack, the victim is often infected only by accessing one malicious domain. (b) In the C&C communication stage, the process of the infected host communicating with the C&C server may not be a text-color ordinary DNS process. The victim host can be connected to the C&C server via an external DNS server.

(6) The Analysis of the Resolution Frequency a Domain Name By a Host In a Period of Monitoring Time: An APT attack can be artificially divided into several stages. Because of the definition of time window in our algorithm, four stages may be grouped into three or two stages. We generate a frequency vector based on the time window in the algorithm, through which the frequency of the host’s resolution of a domain name is described.

### 4.3. The Characteristics of the Relationship between Request Message and Response Message

First of all, we introduce the attack method of using DNS tunnel in the DNS resolution process. The PTR record in the DNS can store almost anything that an attacker wants. An attacker can put the payload in the PTR record and map the IP and domain name. As long as the IP is reversely resolved by the DNS protocol on the attacker’s host, the payload will be received. The most important point is that most firewalls, intrusion detection systems, and situational awareness systems do not audit the DNS protocol, so this traffic is almost never intercepted. The payload is not saved in the file but stored in memory, or it may also bypass the local killing. In the study of Aaron Zimba et al., an attacker successfully uses the external DNS server to obtain the IP of the target host and then attacks through the DNS tunneling. In this process, the target host’s DNS server does not accept any DNS requests. The attacker can get the desired data by reverse analysis. Traditionally, DNS tunneling has been used for command and control [25,26], but it has recently been used by APT attackers with more obvious data infiltration techniques [15,27]. In some attacks, the DNS server that the attacker controls is not online in the long term. It is not available for a victim in the target network to resolve the domain name specified by the attacker, which leads to the unusual DNS resolution. In the case of these attack failures, we can find the infected host by the following feature 7.

(7) The Time Interval Between DNS Response and the Request. There are two reasons for the proposition of the new feature of the time interval between the DNS response and the request. (a) In some of the APT attacks, the resolution process of DNS is unusual, so the feature we propose can reflect the complete 0 resolution process. (b) The access frequency of a malicious domain name should be below a certain threshold. The malicious domain names are not in the cache of the respective level of in the DNS server. The DNS server that caches malicious DNS information can be located by analyzing the interval time, which means that the suspiciousness of DNS resolving on this server is increasing.

(8) The Times That a Domain Name Is Resolved Into Different IPs In a Certain Period of Time: The number of host for the domain was unexceptionally high. It is interesting to note that the number of generated hostnames was way higher than the number of IP addresses supported by the subnet of the Dnscat2 (a malware is often used in APT attacks) server.

## 5. Scoring Algorithm

### 5.1. Method Discussion

In prior works analyzing DNS logs, researchers often use machine learning algorithms and graph algorithms. The purpose of the machine learning algorithm is to assign a weight to the features in the input feature vector and to estimate the extent to which each feature affects the outcome through multiple iterations. Due to the limitations of the classifier method, when the training data reaches a certain scale, the performance of the model trained by machine learning tends to be static. In deep learning, models have high complexity and millions of parameters. On small samples, the number of parameters is much larger than the entire training set, and it is easy to produce overfitting. In other words, the complexity of the neural network at this time made him actually memorize the entire sample without learning and generalization. This phenomenon has been alleviated on large-scale datasets. And the complexity of its model makes it powerful enough to express, which means it is close enough to the real function. We chose to use a deep learning algorithm in this paper. First of all, deep learning not only allows us to study the input feature matrix but also considers the relationship between the features. In the process of deep learning, we use the hidden layer for training the features, which are generated by the previous layer. So, we can reflect the relationship between features and the features between these relationships which we have not considered in the past. Our data volume is very large, reaching 376,605,606. The use of deep learning for data is theoretically superior to machine learning. We designed a deep neural network to learn an effective model to mine the relationship between the DNS activities and APT attacks.

### 5.2. Model Overview

**Figure 3:**
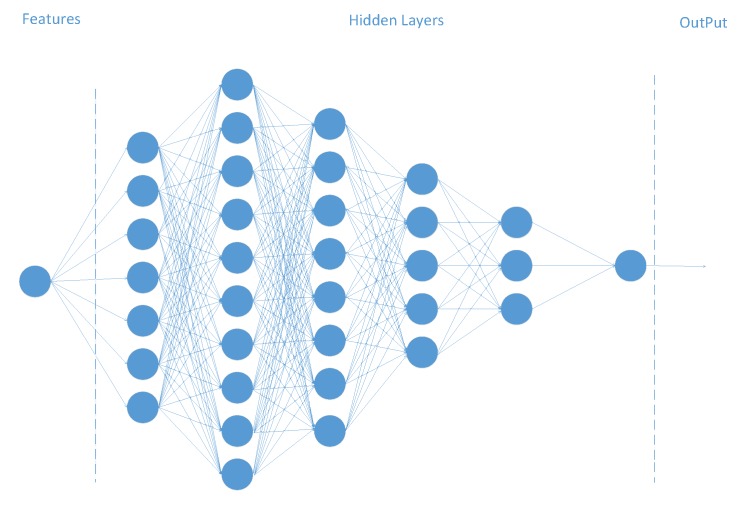
Deep learning model.

In the deep learning algorithm for DNS behavior analysis and evaluation, we generate a 6-layer fully connected neural network with each layer at 7, 10, 8, 5, 3, and 1 (see in Figure 3). The data we input is a set of 8-dimensional feature vectors with serial numbers. After removing the first column of numbers representing the relationship between IP and domain, we get the 7-dimensional vector. So, we generate seven neurons when building the input layer. We want the output to be a one-dimensional label matrix. We believe that the closer the value is to 1, the more suspicious the DNS behavior is.

The data we input is a set of 8-dimensional feature vectors with serial numbers.

(1) $$\delta_{i}=y_{i}\left(1-y_{i}\right)\left(t_{i}-y_{i}\right).$$

In Equation (1), for output node layer *i*, $\delta_{i}$ is the error of the node. $y_{i}$ is the output value of the node. $t_{i}$ is the sample corresponding to the target value of the node.

(2) $$\delta_{i}=a_{i}\left(1-a_{i}\right)\sum_{k\in outputs}w_{ki}\delta_{k}.$$

In Equation (2), for hidden layer nodes, $a_{i}$ is the output value of the node. $w_{ki}$ is the weight of the connection of the node to its next level node *k*. $\delta_{i}$ is the error of the node of the next layer of the node.

(3) $$w_{ji}\leftarrow w_{ji}+\eta \delta_{j}x_{ji}.$$

To update the weight on each connection: In Equation (3), $w_{j}i$ is the weight of node *i* to node *j*. η is the constant of the learning rate. $\delta_{j}$ is the error of node *j*. $x_{ji}$ is the input that node *i* passes to node *j*. Activation Function rectified linear unit (ReLU):

(4) $$f\left(x\right)=\left\{\begin{array}{cc} x, & if\;x\geq 0\\ 0, & if\;x<0 \end{array}.\right.$$

The ReLU can be seen in that, when x<0, ReLU is hard saturated, and when x>0, there is no saturation problem.

For loss function, we use the cross entropy function:

(5) $$H\left(y,a\right)=-\frac{1}{n}\sum_{n}yloga+\left(1-y\right)log\left(1-a\right).$$

In Equation (5), *y* is the desired output, and *a* is the actual output of the neuron. When the error is large, the weight update is fast. When the error is small, the update of the weight is slow. This is a very good property.

## 6. Evaluation

### 6.1. Experimental Data

In data pre-processing phase, there are in total 4,907,147,146 pieces of initial data of 47 days DNS request records of Jilin University Education Network. After DNS data pre-processing, 376,605,606 of these are left, which effectively reduces our time for experiments.

(1) Black Data: In the selection of black data, we refered to a large number of APT attack reports. Samples of available APT attacks were retrieved by reading the attack reports and related papers. According to a report by Kaspersky Lab [28], we define a bunch of DNS traffic in Greece, which reflects that the attacker transfers the IP to the victim’s area via the groundwater server. According to the report published by BAE [29], we find a phenomenon in which an IP corresponds to two different domain names. This report also points out that an attacker has specific working hours, like a human being. This feature is also revealed by a report by Clearskysec [30]. We simulated this feature to some extent. Some malicious DNS traffic is mostly composed of multiple IPs resolved by one domain owner [31]. We also read other reports [32,33] to seek out real APT attack’s details and simulate them. We finally obtained a black dataset of 15,338 by simulation.

(2) White Data: In the white dataset construction of this paper, we used the top one million domain names in the white dataset based on alex rankings for classification. We believe that the access log of the accesses to the domain names in this whitelist is innocent.

(3) Grey Data: During the pre-processing stage of the data, our original data is 47 days of DNS records from Jilin University Education Online, a total of 4,907,147,146. After filtering through the pre-processing of the number of visits and whether the domain name is within the whitelist, 376,605,606 are finally left, which effectively reduces our time of experiments. Due to the difficulty of obtaining black data, over-sampling problems have occurred in our training dataset. To solve the noise caused by oversampling, we used the integrated learning strategy of stacking. The experimental results mentioned below are the result of using the stacking strategy.

### 6.2. Experiment Platform Description

We used the Win10 system Python3.5 IDEL environment to process the data. In order to use the deep learning system of tensorflow, we used Nvidia GTX 1080TI as the graphics card. In the data pre-processing stage, we needed to read a large amount of data for text analysis, so we selected E5-2650v4 on the CPU and used four channels of 64 gb ddr4 3000 hz for memory(see in Table 2).

### 6.3. Analysis of Experimental Data

After pre-processing, in order to carry out deep learning analysis on our data, we feature vectorized pre-processed data, and the data of each dimension has the following explanation:

Feature1

Length of domain name. We believe that the length of the domain name is randomly generated by the attacker. A large amount of data can reflect some characteristics of the attacker’s naming. Since the maximum length of the domain name is 63, we define

$Feature1=\frac{domain\_length}{63}$.

The domain_length means the length of the domain name.

Feature2

Number features in domain name. We define

$Feature2=\frac{domain\_number}{domain\_length}$. The domain_number means the the number of digits in the domain name.

Feature3

Keyword features in the domain name. We build two sets when generating this feature: famous and wordlist. Famous set contains the famous domain names and functional vocabulary. We define

Feature3=sizeof(famous∩wordlist).

Feature4

The time of continuously resolve the same domain name in monitoring time. We believe that the resolution of the same domain name in θ seconds is continuous. We define

$Feature4=\frac{request\_count}{\theta }$.

Feature5

Whether the resolved domain name only appears once. We define

$Feature5=\left\{\begin{array}{cc} 1, & if\;request\_count=1\\ 0, & if\;request\_count=0 \end{array}\right.$.

The request_count means the request time of a same domain name.

Feature6

Host frequency analysis of a domain name in monitoring time. We define a time window λ and generate a frequency vector *f* within the detection time based on λ.

We define f=[f1,f2,f3,f4,f5,f6,f7,f8].

The f_i,i∈[1,8] means the frequency in time windows. We define the interval between non-zero elements in the frequency vector as ε. We define $Feature6=\frac{a}{request\_count}$.

Feature7

Feature of the relationship between DNS request behavior and response behavior. We define Feature7 has a value of *responsetime-requesttime* if responsetime exists; otherwise, the value is 1.

Feature8

Feature of the the numberof times a domain name is resolved into different IPs in a short period of time. We define $Feature8=\frac{ip\_count}{resolve\_frequence}$. ip_count indicates the number of IPs resolved by one domain name. resolve_frequence indicates how often a domain name is resolved. We define $resolve\_frequence=\frac{resolve\_time}{total\_detection\_time}$. resolve_time refers to the number of times a domain name is resolved, and total_detection_time refers to the detection cycle of the entire system.

### 6.4. The Description of Model Training Results

Through the training of the model, our fully-linked neural network can score the malicious degree of one DNS behavior, and its value is in the interval of [0,1]. With the dataset we currently have, we define a malicious threshold of 0.95, which means that when the score is higher than 0.95, we believe that this DNS behavior is malicious.

### 6.5. Model Training and Results

After completing the model training of the full-linked neural network, we mixed the 15,338 pieces of black data mentioned above with the gray dataset in which data has been pre-processed. After getting the results from the application through our assessment of system of the new fully-linked neural network, we found 40,758 suspicious DNS behavior records containing the simulation data we put in. Our algorithm obtained new suspicious DNS behavior; thus, our method is considered effective.

We used three standard evaluation parameters to evaluate the test framework we designed. The evaluation parameters include: FP, Recall, Accuracy Rate, etc. We introduce a confusion matrix to show the relationship between false positive rate, false negative rate, malicious sample detection accuracy, and normal sample detection accuracy. These relationships are detailed in Table 3. FPR (FP-rate) indicates the proportion of malicious DNS behavior which is wrong (that is determined by our detection framework to be malicious) to all DNS behavior. Recall indicates the proportion of the malicious acts (that the detection framework determines to be malicious) to all DNS behavior. The following formula shows the intrinsic relationship between these evaluation parameters.

(6) $$FPR=\frac{FP}{FP+TN},$$

(7) $$Recall=\frac{TP}{TP+FN},$$

(8) $$Accuracy=\frac{TP+TN}{TP+TN+FP+FN}.$$

In order to further determine the stability of model training, we divide the training set into ten parts and use 10-fold cross validation. The results show that the model’s average accuracy is about 97.6 %, average recall is about 96.8%, and average FPR is about 2.3%.

Table 4 shows the accuracy, the true positive rate, and the recall of our detection.

**Figure 4:**
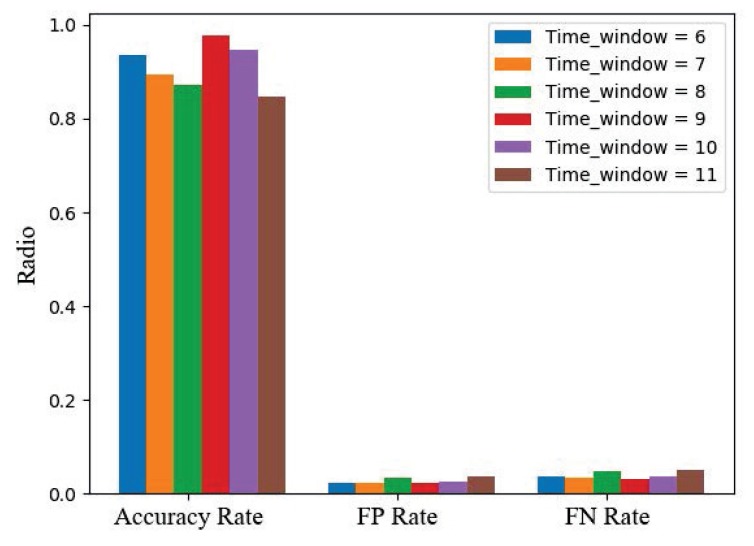
Detection results comparison of different time windows.

In order to judge the rationality of our parameter selection, we up-regulate and down-regulate the parameters of the time window(see in Figure 4) in the feature vector. We also attempt to change the learning rate in a model of a fully linked neural network. First, we adjust the time window between 6 and 10. We select the appropriate time window based on the accuracy rate. We find out that the time window at the value of 9 would be appropriate. After the reprocessing of the data, the experiment results show that the false positive rate is about 2.3%, the false negative rate is about 3.1%, and the accuracy rate is 97.6%. We find that after this parameter adjustment, the false positive rate has a slight increase, and the false negative rate just changes a little.

Then, we adjust the learning rate η. We use a fixed learning rate and an exponential decay learning rate algorithm. After multiple comparisons, we find that, when we use the exponential decay learning rate algorithm and when we use the parameters with the learning rate set at 0.1 and for which every 100 rounds of learning are multiplied by 0.96, the training model can be built more quickly when the loss is similar to the fixed learning rate.

### 6.6. Comparison with Different Feature Sets

**Figure 5:**
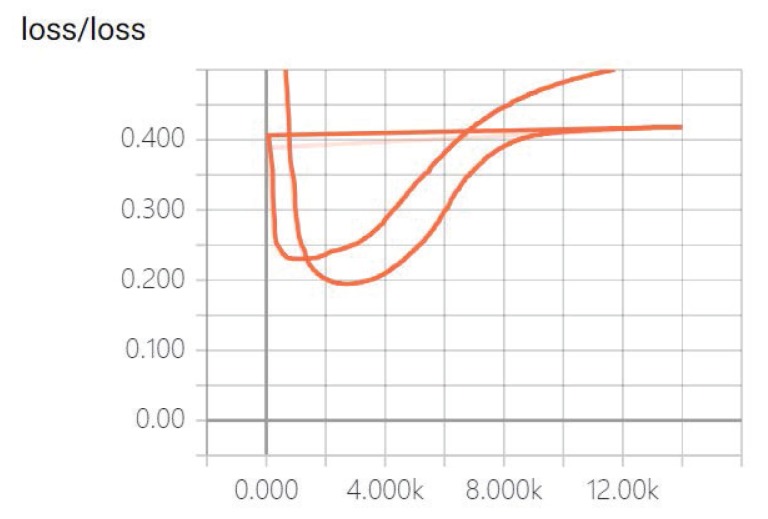
Different feature sets comparison result on loss.

To prove the correctness of the new features, we used the old feature set (without * features) and the new feature set (joined * features) for training. We can see that our model has a smaller loss rate after adding new features (see Figure 5). The loss is used to estimate the degree of inconsistency between the predicted value of the model and the real Y. It can be seen that, the lower the loss, the higher the accuracy of the model.

**Figure 6:**
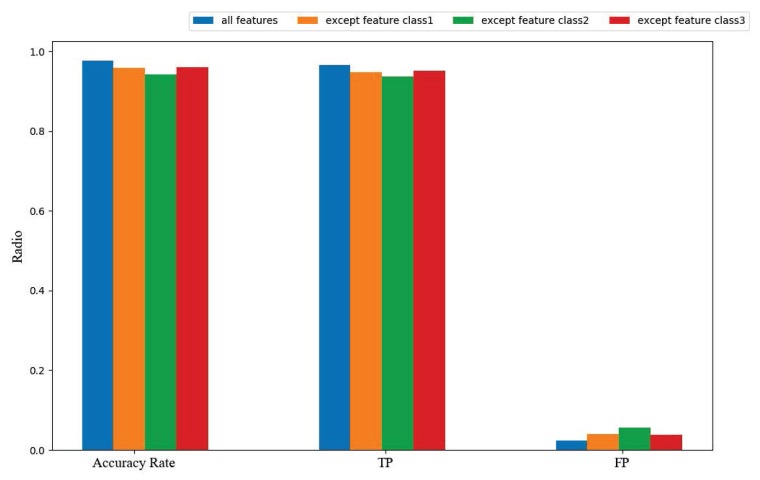
Different feature sets comparison result on Accuracy Rate, true positive (TP), and false positive (FP).

In Figure 5, the x-axis represents the number of iterations of the training and the y-axis represents the loss rate of our model. In order to further determine the validity of our feature set, we eliminated three feature sets consisting of the feature classification methods proposed in Section 4 to perform verification experiments. The experimental results, as shown in Figure 6, demonstrate that the features we propose are valid.

### 6.7. Comparison with Previous Work

In the ‘IDNS’ system of Zhao et al. [9], the authors used the J48 decision tree algorithm to classify the data after the model is constructed. The authors proposed 14 features about DNS to find the suspicious traffic. None of the 14 features reflected the relationship between the DNS request message and response message, which makes it impossible to detect certain abnormal behavior of special contacts when performing the classification algorithm. In the feature of ‘IP in the Same Class B Range of Known C&C Servers’, the authors mention that there are many C&C servers in the same Class B IP addresses range and even in the same Class A range. Although we agree with the authors’ ideas, we can get more accurate and effective results if combining the authors’ idea with the relationship between our request message and response message. The authors used the IEEE J48 C4.5 algorithm to classify DNS features and then combined the classification with the scores after the analysis of the IP stream. We believe that, if we use our method to achieve the same accuracy as that of the method of using IP stream in ‘IDNS’, our method is effective. In this paper, the authors divided the features into three vector groups. The three feature vectors will be concatenated into one feature vector V(ipi), which will be fed into the trained reputation function. What we needed to compare is the malicious DNS classifier and our rating system. Although we were unable to obtain the dataset of the above researchers, we conducted the following experiments in order to compare the performance of different classification methods on the same dataset that we mentioned in Section 6.1. We compared the decision tree framework of J48 c4.5 with the deep learning system we built. In order to better reflect the usability of the features proposed in this paper, we also conducted comparative experiments on different feature sets.

Exp 1 (see in Table 5) represents the experimental results of the deep learning framework using the new features, Exp 2 represents the experimental results of the J48 c4.5 framework using the new features, Exp 3 represents the experimental results of the deep learning framework using the old features, and Exp 4 represents the J48 c4 using the old features and J48 c4.5 framework experimental results. We have seen through the experiment results that the introduction of new features has improved the accuracy of machine learning algorithms and deep learning algorithms, to some extent. The accuracy relationship between the machine algorithm using the new features and the deep learning algorithm using the old features cannot be determined, which is caused by the number of datasets we train and the number of different features. We expect to work or to improve in the future, as well as make our method more effective.

### 6.8. Analysis of Experiment Results

Experiments prove that the proposed method is flexible and scalable. The parameter setting has a great influence on the experiment results, and the parameter setting must be strictly controlled according to the existing related APT reports. Since the entire system is based on features, new features can be quantified and added to the system to improve detection accuracy and efficiency.

## 7. Conclusions and Future Work

The number of APT attacks in the current network environment is enormous, and the complexity and destructiveness are constantly improving. This research developed a novel deep-learning-based detection system to detect malicious DNS behavior. The detection system of this paper is divided into the following parts: DNS data collector, DNS data pre-processing, DNS behavior feature extraction, and DNS behavior analysis and evaluation (deep learning algorithm). Data pre-processing and feature extraction associate DNS logs with APT attack behavior to ensure to reduce false positives. Through DNS behavior analysis, we evaluate and analyze the data by the most accurate and efficient model we can achieve. The experiment results show that the accuracy of our detection system has reached 97.6%. However, our feature algorithm also makes some normal DNS access traffic increase the suspiciousness of white data because it is in the same DNS server with the malicious domain name or because its resolution time is similar to that of suspicious domain names. This is also the optimization goal we need to consider in the future. For future work, some improvements can be made within the system. After a measurable assessment of DNS behavior, we can get suspicious domain names and hosts. For these objects, we can use a feedback mechanism to upgrade our detection system. It would be beneficial to use our detection system on more real APTs, but due to the difficulty of obtaining samples, we need to do more work.

## Figures and Tables

**Table 1:** Feature sets (* for new features).

Feature Set	No.	Feature Name	
Domain Name-based Features	1	Length of domain name	
	2	Number features in domain name	
	3	Keyword features in the domain name	
Feature of the Relationship between DNS Request Behavior and Response Behavior	4	Time of continuously resolve the same domain name in monitoring time	
	5	Whether the resolved domain name only appears once	
	6	Host frequency analysis of a domain name in monitoring time	*
Feature of the Relationship between DNS Request Behavior and Response Behavior	7	Time interval between the DNS response and the request	*
	8	Host frequency analysis of a domain name in monitoring time	*

**Table 2:** Experiment platform.

DEVICE	Detailed Specifications
CPU	E5-2650v4
GPU	Nvidia GTX 1080TI
memory	4 channels of 64 gb ddr4 3000 hz
OS	Ubuntu 18.04.2 LTS
environment	Python3.5, CUDA 9.0.176(6.14.13.8554)
	tensorflow 1.8.0

**Table 3:** Confusion matrix.

Predicted Class			
Actual class		True	False
	True	true positives	false positives
	False	false negatives	true negatives

**Table 4:** Result of experiment.

ACCURACY	RECALL	FPR
97.6%	96.8%	2.3%

**Table 5:** Comparison result data.

No.	ACCURACY	RECALL	FPR
Exp 1	97.6%	96.8%	2.3%
Exp 2	96.1%	95.2%	3.8%
Exp 3	95.9%	94%	4%
Exp 4	94.3%	93.8%	5.6%

## Abbreviations

The following abbreviations are used in this manuscript:

MDPI	Multidisciplinary Digital Publishing Institute
DOAJ	Directory of open access journals
TLA	Three letter acronym
LD	linear dichroism
